# Breeding transients in capture–recapture modeling and their consequences for local population dynamics

**DOI:** 10.1038/s41598-020-72778-x

**Published:** 2020-09-25

**Authors:** Daniel Oro, Daniel F. Doak

**Affiliations:** 1 IMEDEA (CSIC-UIB), 07190 Esporles, Spain; 2 Environmental Studies Program, University of Colorado, Boulder, Boulder, CO 80309 USA; 3 CEAB (CSIC), 17300 Blanes, Catalonia, Spain

**Keywords:** Biological techniques, Ecology

## Abstract

Standard procedures for capture–mark–recapture modelling (CMR) for the study of animal demography include running goodness-of-fit tests on a general starting model. A frequent reason for poor model fit is heterogeneity in local survival among individuals captured for the first time and those already captured or seen on previous occasions. This deviation is technically termed a transience effect. In specific cases, simple, uni-state CMR modeling showing transients may allow researchers to assess the role of these transients on population dynamics. Transient individuals nearly always have a lower local survival probability, which may appear for a number of reasons. In most cases, transients arise due to permanent dispersal, higher mortality, or a combination of both. In the case of higher mortality, transients may be symptomatic of a cost of first reproduction. A few studies working at large spatial scales actually show that transients more often correspond to survival costs of first reproduction rather than to permanent dispersal, bolstering the interpretation of transience as a measure of costs of reproduction, since initial detections are often associated with first breeding attempts. Regardless of their cause, the loss of transients from a local population should lower population growth rate. We review almost 1000 papers using CMR modeling and find that almost 40% of studies fitting the searching criteria (N = 115) detected transients. Nevertheless, few researchers have considered the ecological or evolutionary meaning of the transient phenomenon. Only three studies from the reviewed papers considered transients to be a cost of first reproduction. We also analyze a long-term individual monitoring dataset (1988–2012) on a long-lived bird to quantify transients, and we use a life table response experiment (LTRE) to measure the consequences of transients at a population level. As expected, population growth rate decreased when the environment became harsher while the proportion of transients increased. LTRE analysis showed that population growth can be substantially affected by changes in traits that are variable under environmental stochasticity and deterministic perturbations, such as recruitment, fecundity of experienced individuals, and transient probabilities. This occurred even though sensitivities and elasticities of these parameters were much lower than those for adult survival. The proportion of transients also increased with the strength of density-dependence. These results have implications for ecological and evolutionary studies and may stimulate other researchers to explore the ecological processes behind the occurrence of transients in capture–recapture studies. In population models, the inclusion of a specific state for transients may help to make more reliable predictions for endangered and harvested species.

## Introduction

Capture–mark–recapture modeling (CMR) is a powerful method to obtain reliable estimates of vital rates in the study of animal demography. In ecological and evolutionary studies, vital rates such as survival, fertility, and dispersal are crucial for explaining and predicting population dynamics, as are trade-offs between some of these rates. In the early 90s, Lebreton et al.^1^ summarized how individual CMR uni-state models can be used to estimate survival and test biological hypotheses concerning the external drivers (e.g., climate) and internal factors (e.g., age, sex) affecting this vital rate. In a few cases, these models have been used to test hypotheses about how reproduction may represent a survival cost and under which environmental conditions these costs may be higher. For instance, Viallefont et al.^2^ attempted to estimate not only survival, but also the costs of reproduction in a colonial bird by using the recapture rate as a surrogate for these costs. Results showed that animals were less likely to be recaptured 1 year after their first breeding attempt than on later seasons, so there was a cost of present reproduction on the future probability of breeding. Other avian studies having recapture rates close to 1 and not using CMR techniques have also found that many birds (in some cases up to 30%) failed to appear in the year following their first breeding attempt^3,4^. More generally, many uni-state CMR models have detected the occurrence of a high proportion of marked animals that do not return to the study area following first breeding attempts^5^.

Technically, these non-returning animals are termed “transients”^6^: individuals that once marked are never recaptured again because they die or disperse permanently from the study area. The term “transient” was originally coined to describe an animal that is caught and marked while it crosses the study area only once, for instance migrating individuals compared to resident individuals. However, “transients” can also include formerly resident animals that permanently emigrate and also animals that die, including those that perish due to the costs of first reproduction^5^. In some cases where the cause of transients is clear, the proportion of transients in uni-state CMR models can be a suitable tool to quantify costs of first reproduction e.g.^7,8^. But in most cases it is not possible to disentangle whether the lower local survival of transients corresponds to permanent dispersal, lower survival after first marking, which often corresponds to first breeding, or a mixture of the two. In addition, dispersal too may represent a cost of breeding^9^, if survival of dispersers is lower or their future breeding success is reduced in a new location. Indirect evidence of the costs of dispersal comes from studies showing that migration is costly (especially for young and inexperienced individuals) compared to individuals that stay at breeding sites^10–12^. However, some individuals may also increase or maintain their reproductive performance after dispersal^13,14^, although estimating the costs and benefits of dispersal is challenging in ecological studies^15,16^.

Regardless of the ecological process behind the occurrence of transients, i.e. lowered survival or permanent dispersal, the existence of transients will affect the dynamics of local populations. First, lower survival may lower individual fitness due to a survival-reproduction trade-off. Even though there are a large number of empirical evolutionary studies on survival-reproduction trade-offs in animals e.g.^15–17^, and some of them used uni-state CMR e.g.^7,8^, relatively little is known about their potential effects on population dynamics. What is known is that the survival-reproduction trade-off is stronger during first-time breeding, since lack of breeding experience may imply a higher survival and a higher selection filter^17–20^. Especially in long-lived species, this cost can be substantial, since individuals reproducing for the first time tend to both produce fewer offspring and pay higher survival costs^21,22^, i.e. it penalizes lifetime reproductive success. Some studies^18,19,23^ have also highlighted the importance of understanding the role of transients in population dynamics at a large spatial scale, when transients are dispersers. Furthermore, transients can show temporal variability depending on environmental conditions; for instance, perturbations affecting habitat suitability (e.g., availability of sheltering sites, density of predators) can increase transients due to permanent dispersal^24,25^, and this should be most pronounced when density-dependence is strong^26^. For instance^27^, suggested that in high density patches, the number of transient *Salamandrina perspicillata* salamanders should play an important role in population size regulation. Spatial heterogeneity in habitat quality also generates variability among populations in the occurrence of transients at large spatial scales^28,29^.

In spite of the potential importance of the processes that generate transients in CMR models, the use of analyses that quantify transients to gauge these effects is still underappreciated^5^. The only way to completely disentangle the biological process behind the occurrence of transients (either permanent dispersal or survival costs after first reproduction) is to have capture–recapture data at large spatial scale. This type of study allows researchers to interpret correctly the process at play, but these studies are rare due to the difficulties at collecting individual capture–recapture data in several patches. However, a comparative approach can still allow useful interpretation of transient estimates in the absence of these ideal study designs. Here, we first review how often the ecological processes behind transients in simple, uni-state CMR models may appear unnoticed or may be confounded by some other processes in those studies. Second, to illustrate these issues and ways to use and interpret transient estimates, we analyze a long-term data set on a long-lived seabird, in which previous studies show that transients appear in high proportions. Next, we show how transients can be incorporated into population models to assess their impact on the temporal dynamics of local populations. Our overall goal is to improve the understanding of transient phenomena and the analysis of its effects on individual fitness and population dynamics.

## Results

**Figure 1:**
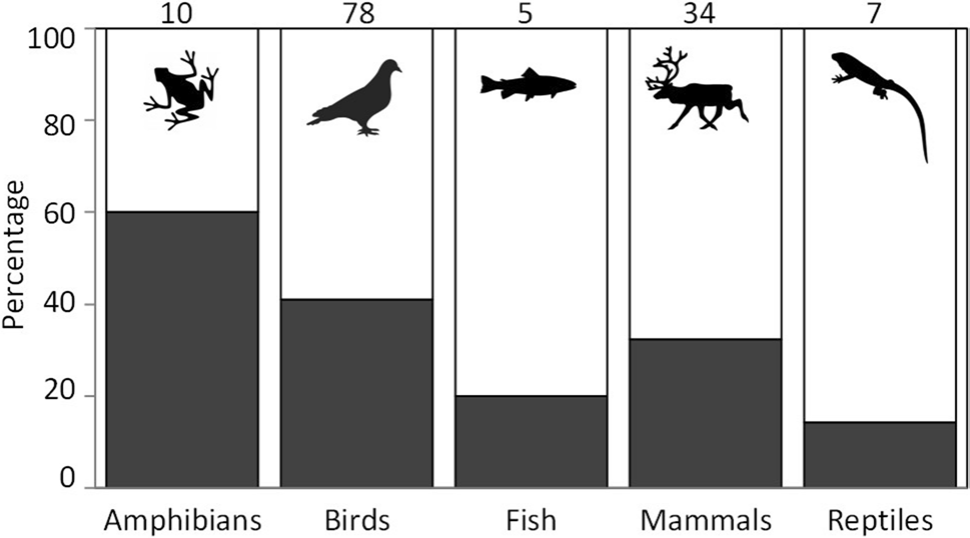
Percentage of transient occurrences in CMR studies (black bars) depending on taxonomic group. Numbers over the bars show the number of species·populations·studies found during the search. Some studies included more than one species. The two studies on invertebrates are not shown.

Of 980 papers examined, 115 fit our criteria, corresponding to 136 different species and populations. These papers include analyses of all major vertebrate groups and a few invertebrates (insects, molluscs). We found that 37% of studies detected transients. The occurrence of transients was higher in amphibians and relatively low for the more mobile taxa (fish and birds), even though they are stronger candidates for having classical transients, in the form of animals crossing a study area only once (see Fig. 1).

Strikingly, more than half of the uni-state CMR studies finding a transience effect did not provide a biological explanation for its occurrence (see Table 1, Table S1).

**Table 1:** Interpretations of the ecological process causing a transient effect when detected by test3.SR goodness-of-fit in CMR modelling for different taxa.

Taxa studied	Biological process proposed	% of studies	Example references
Shearwaters, petrels, flamingos	Cost of first reproduction	3.8	^7,24,28^
Shearwaters, auklets, skuas	Prospecting	5.8	^101–103^
Tigers, turtles, passerines	Transients over the study area	9.6	^104,105^
Beetles, newts, salamanders, turtles, aprons, bats, terns, gulls, penguins	Permanent dispersal	21.2	^27,92^
Toads, passerines, guillemots, voles	Age effect	7.7	^106,107^
Cottonmouths, seals, bears, voles, mice, manatees, bats, swallows, eiders, terns, waders, albatrosses, shearwaters, fulmars, petrels, penguins, toads	Not interpreted; only in some cases there was an explicit statement about the presence of transients	51.9	^108^

The percentage column shows how often a biological process was proposed when transients were found from a sample of 52 studied populations (all species, but not all studies, are referenced). Methodological issues and a complete table with all references can be found in Table S1.

Table S1 shows a list of papers finding transients in uni-state CMR studies and the biological processes or methodological biases proposed to explain the occurrence of these transients. The most common explanation in papers detecting transients (21% of cases) was that they corresponded to permanent dispersal (without considering that this may have occurred as a cost of reproduction), while the rest of explanations were that transients were real transient individuals through the study area, an effect of age, or they occurred because there were animals prospecting the reproductive area. None of the studies contemplated the potential occurrence of multiple processes. Only three studies, working on several species of burrowing seabirds (in which only breeding individuals were marked and recaptured) and on flamingos, stated that transients may correspond to survival costs of reproduction for first-time breeders, in particular after adverse environmental conditions^7,30,31^ (see Table S1).

**Figure 2:**
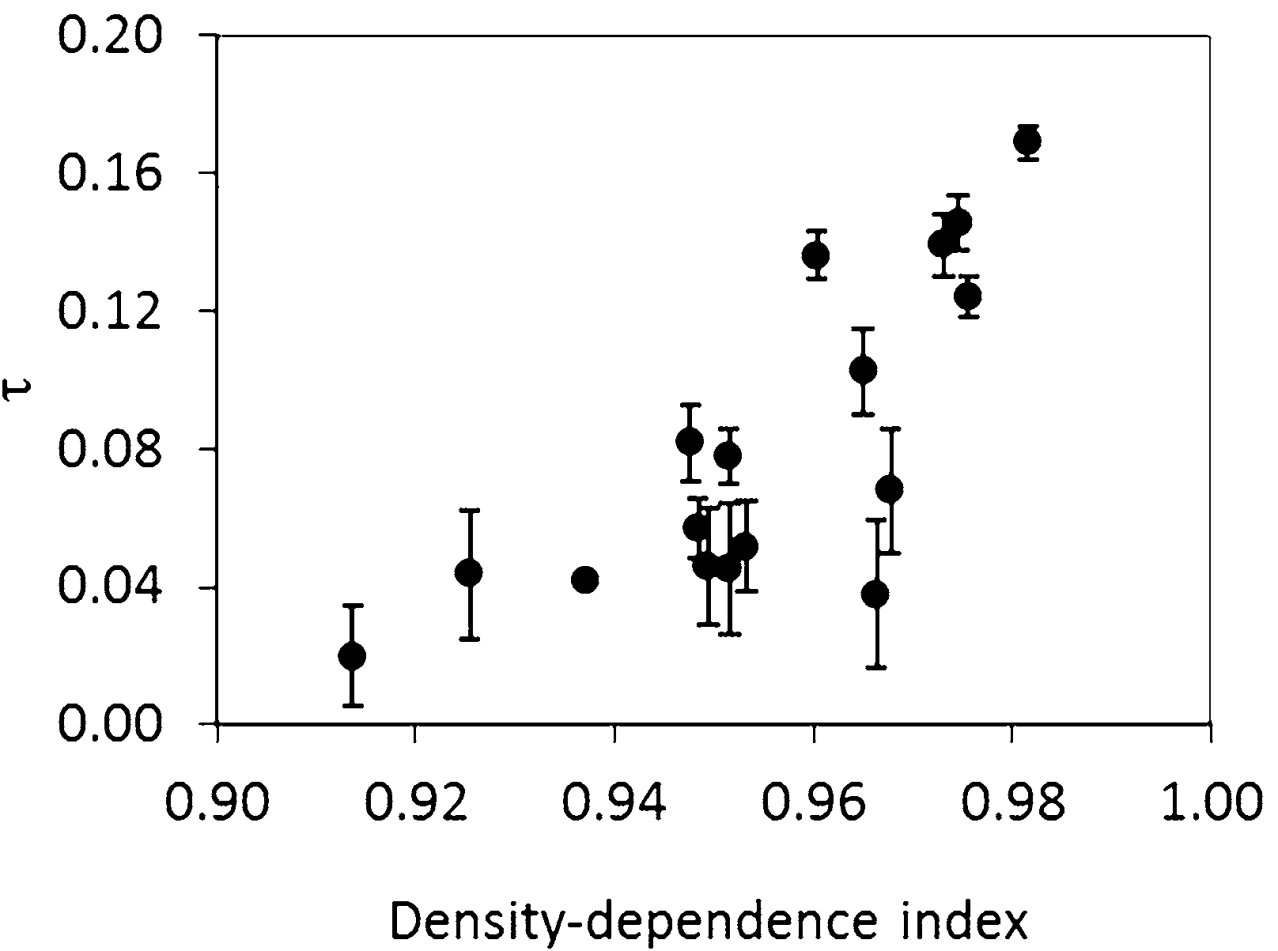
Association between the proportion of transients τ (mean and 95% confidence intervals CI) and the strength of density-dependence over the Audouin’s gull study, calculated as food availability per capita. The density-dependence covariate explained 56% of the variability in transient occurrence. As an example here, we took the transients for individuals breeding first at 4 years old (the modal age of first reproduction in an average year), and the selected model also showed that transients increased with age (Table S3).

Transient CMR modelling in Audouin’s gulls (see details in Table S2) showed that transient loss varied with time and differently for each age-class (model 1 in Table S3). The percentage of deviance explained by the model with a total density-dependence covariate was 56% (ANODEV analysis, see model 6 in Table S3). Much of the unexplained variance may be generated by the impact of terrestrial carnivores entering the colony in the last 15 years, which caused additive mortality^32–34^. The models show that the proportion of transients increased with age at first breeding, and it was also affected by density-dependence (Table S3, see also Fig. 2). For instance, for gulls breeding for the first time at 4 years old, the proportion of transients ranged from 0 to 17%, whereas for individuals recruiting at > 6 years old, this proportion was substantially higher, ranging from 37 to 81%.

When the strength of density-dependence increased and environmental conditions became harsher, all survival and reproductive rates decreased (particularly recruitment γ) and the apparent local survival costs of first reproduction greatly increased (Table 2).

**Table 2:** Sensitivities and elasticities of population growth rate of Audouin’s gull compared to life table response experiment (LTRE) contributions of model parameters of the life cycle shown in Fig. 5.

Parameter	Parameter value		Δ*p*	Elasticity	Sensitivity	LTRE contribution
	Good season	Bad season				
ϕ_1_	0.919	0.653	− 0.265	0.039	0.078	− 0.019
ϕ_2_	0.867	0	0.079	0.055	0	
ϕ_A_	0.976	0.912	− 0.064	0.849	0.968	− 0.061
**τ**_***i***_						
τ_3_	0.001	0.022			− 0.021	
τ_4_	0.140	0.150			− 0.020	
τ_5_	0.189	0.446			− 0.010	
τ_6_	0.277	0.616			− 0.003	
τ_7_	0.441	0.853			0.000	
τ_total_			1.038	− 0.083		− 0.063
**γ**_***i***_						
γ_3_	0.389	0.198			0.008	
γ_4_	0.483	0.328			0.014	
γ_5_	0.369	0.392			0.008	
γ_6_	0.201	0.130			0.009	
γ_7_	1.00E−09	1.00E−09			0.005	
γ_total_			− 0.394	0.078		− 0.045
*F*	0.501	0.212	− 0.289	0.074	0.129	− 0.044
*F*′	0.262	0.102	− 0.160	0.005	0.009	− 0.002
*λ*	1.0889	0.9445	− 0.144			

Δ*p*_*i*_ = difference in parameter *i* between estimates for the best and worst season of the study. To simplify the interpretation and comparisons between demographic rates and their LTRE contribution, only the total values for the proportion of transients (τ_total_) and recruitment (γ_total_) are shown. Elasticities correspond to the matrix using parameters for the good season; sensitivities come from the mean matrix between the good and the bad season^96^. Values of λ for the good and bad seasons and their difference are also shown. *F* and *F’* are the fertilities (as fledglings per breeding pairs) for resident and transient individuals respectively. ϕ_1_, ϕ_2_, ϕ_A_ are survival for 1 year, 2 years old and adult breeders respectively; τ_*i*_ and γ_*i*_ correspond to the age parameters for transients and recruitment respectively (Table S2; the rest of estimates come from previous and unpublished studies, see “Methods”).

**Figure 3:**
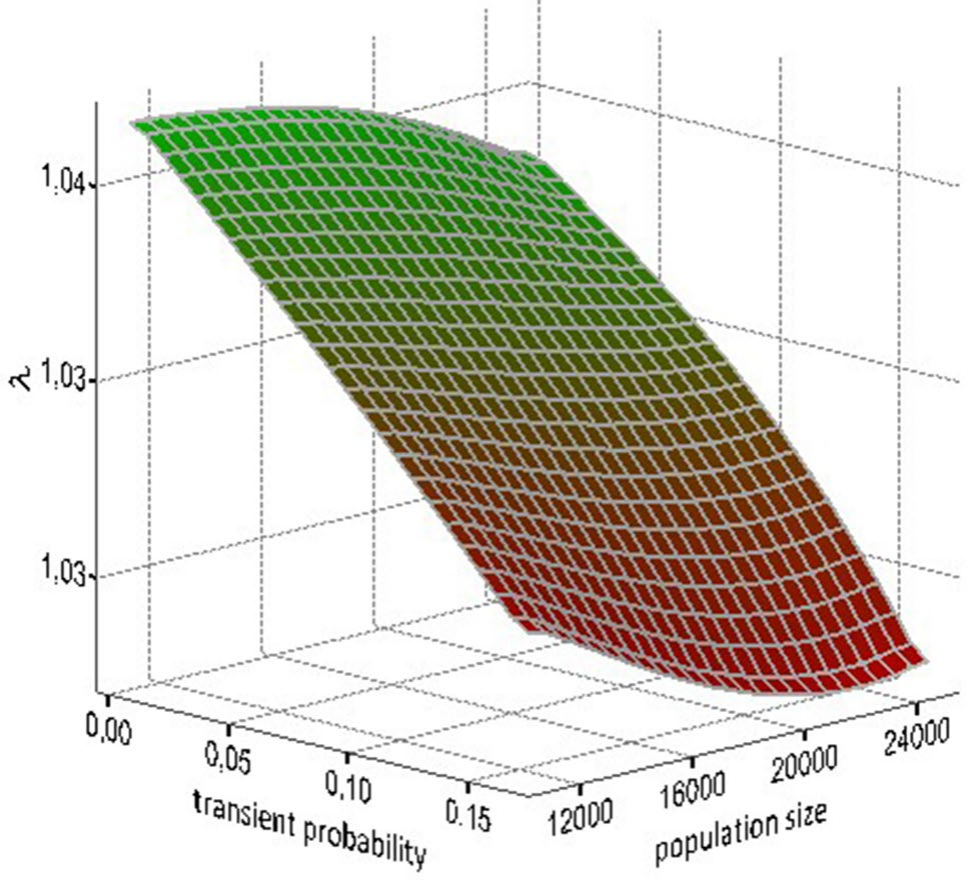
Variation of population growth rate (λ) as a function of population density and the intensity of transient probability (for individuals aged 4) as an indicator of reproductive costs in Audouin’s gull.

When we ran our population model with different values of population size and transients, we found that both variables had a negative influence on population growth rate (Fig. 3).

**Figure 4:**
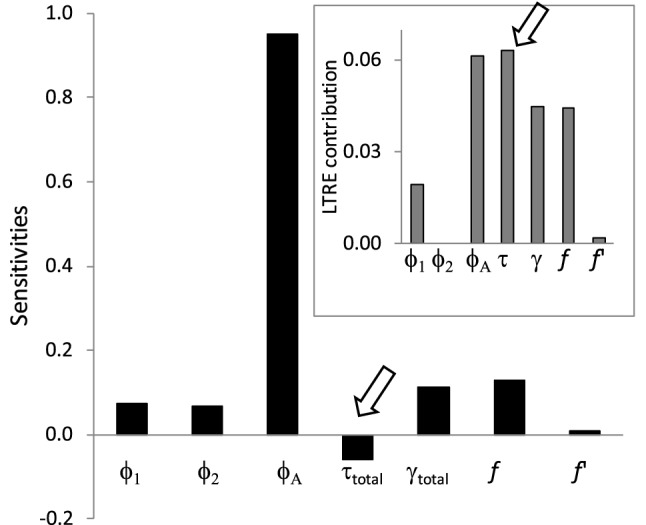
Sensitivities (from a matrix model using mean values of vital rates) and LTRE contributions (in absolute value, inset panel) of vital rates on population growth rate of the local population dynamics of Audouin’s gulls (see Fig. 4). Arrows point to the values of sensitivities and LTRE contributions for total proportion of transients considering all age-classes.

**Figure 5:**
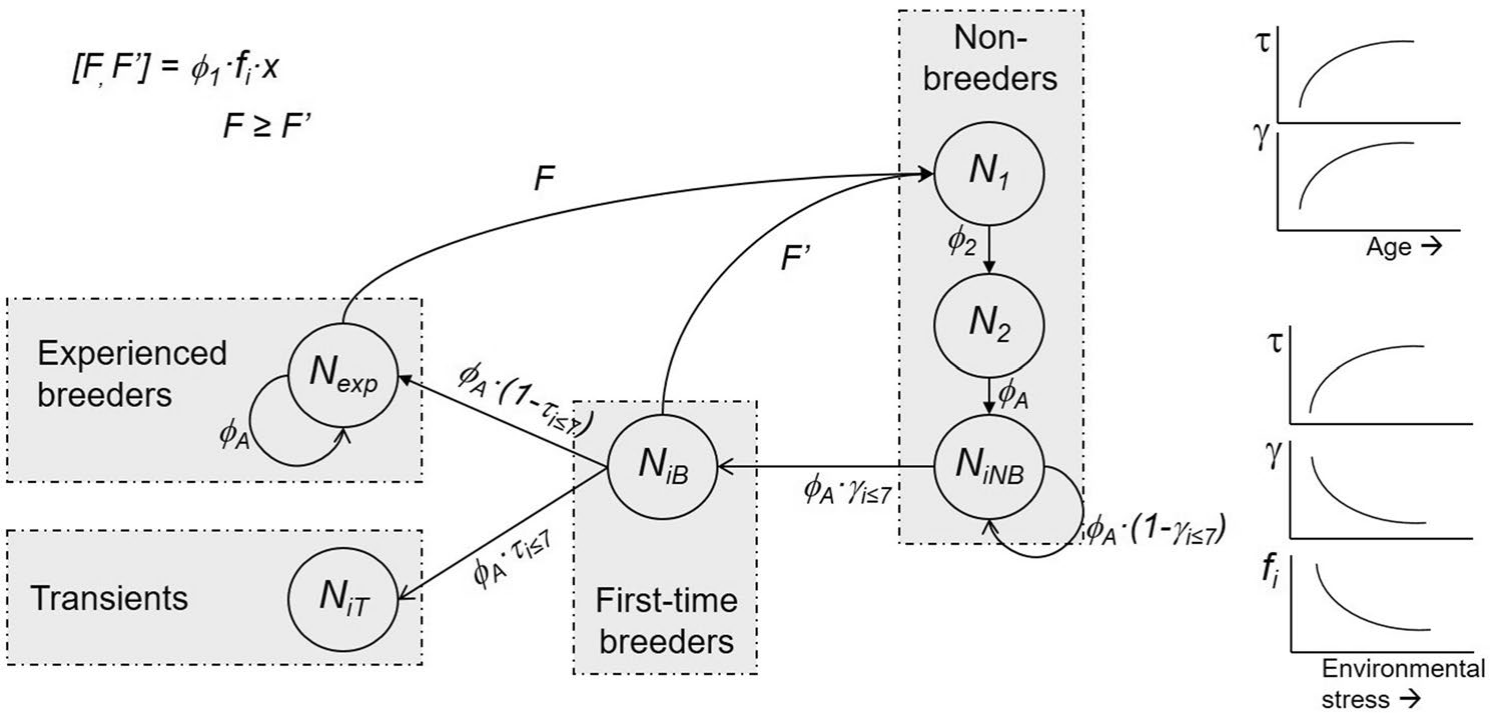
Life-cycle and structure of the state-based population model for the case study (Audouin’s gull), assuming a pre-breeding census. Transitions between stage classes (nodes) occurred over the time of 1 year and are indicated by arrows, each of which contains the probability of individuals to move or contribute to the next node at the end of the arrow. Nodes *N*_*1*_ correspond to 1 year old individuals, *N*_*2*_ to 2 years old, *N*_*iB*_ to breeding individuals of age *i*, *N*_*iNB*_ to non-breeding individuals of age *i* and *N*_*iT*_ to first-time breeders of age *i* that pay a cost of reproduction; *i* ranges from 3 to 7. How transients, recruitment and fertility changed with age and environmental stress (density-dependence) is also shown. ϕ_1_, ϕ_2_ and ϕ_A_ are survival for 1 year, 2 years old and adult animals respectively; γ_*i*_ are the probabilities of first breeding at age *i* (3 ≤ *i* ≤ 7); τ_*i*_ are the probabilities of being a transient for individuals first breeding at age *i*; *F* and *F*’ are fertilities for experienced and first-time breeders respectively; *x* is the sex-ratio, which was set to 0.5.

As expected, sensitivities and elasticities confirmed that population growth is particularly sensitive to changes in vital rates with very low variability, in particular adult survival (Table 2). Nevertheless, LTRE analysis showed that population growth can be substantially affected by changes in other traits that are more variable under environmental stochasticity and deterministic perturbations, such as recruitment, fecundity of experienced individuals, and transient probabilities (Table 2, Fig. 4). Indeed, the summed effect of changes in transient return probabilities was greater than variation in adult survival in shifting population growth across contrasting conditions (Fig. 5).

## Discussion

We show here that transients can be quantified in many uni-state CMR studies. Why transients occur more frequently in amphibians and less for the more mobile taxa (fish and birds) is unknown, but this may be biased by the field protocols commonly used for different taxa, as well as how easily individuals of different ages (and particularly younger individuals, who often have lower survival^35,36^) are caught. Despite the high occurrence of transients, results of these analyses are rarely considered as a way to explore or quantify the ecological processes behind the occurrence of transients. This is so even in studies finding transients considering a two-ages classes in the starting model, when newborns were included in the analysis^14,37^, or in studies working only with breeding adults^19,38^. While mortality after first reproduction is a direct fitness cost, permanent dispersal is not necessarily a cost to an individual, but may still have consequences to the local population growth rate. These consequences depend on the rate of permanent dispersal between local populations and its symmetry. Unfortunately, uni-state CMR studies are not in most cases able to distinguish dispersal from mortality, although multi-state models and data on multiple populations can in some cases allow this distinction e.g.^29,39,40^ (Table S4). However, a few studies working at large spatial scales actually suggest that transients correspond to survival costs of first reproduction and not to permanent dispersal^13,14,29,39^. For instance, in a capture–recapture study on slender-billed gulls (*Larus genei*) comprising 45 different colonies, survival was modeled considering two-age classes (juveniles and older birds), because all histories started when individuals were chicks, and a lower survival for juveniles was expected and found in the models^14^. However, the test 3.SR was still very strong when the first encounter was deleted from the capture–recapture histories, suggesting that many birds payed a survival cost at the start of breeding. Other studies working with extremely philopatric species, such as Procellariiforms, also suggest that the presence of transients corresponds to survival costs of first reproduction. Albatrosses, shearwaters and petrels show relatively high natal dispersal, but very strong philopatry once they start breeding in a site^41^. Most capture–recapture studies performed on breeders in these species showed the presence of transients^30,31,42–49^ and in some cases they have even been explicitly treated as survival costs of first reproduction^18,20,50^. Many of these study cases correspond to birds. Species that are more mobile should be more prone to show transience in the form of permanent dispersal after the first resight. Nevertheless, we have also shown, despite the challenges imposed to disentangle the biological drivers generating transients, a few studies on mobile species demonstrate that at least part of the transients are caused by a cost of the first reproduction. This also means that studies finding transients on species that have low mobility should consider a fortiori option of a survival cost of first reproduction.

We also show how biological hypotheses related to the presence of transients can be tested. For Audouin’s gulls, individual covariates such as age as well as environmental stress, such as strength of density-dependence for food, influenced time-varying proportion of transients. As we noted above, multi-site CMR studies on Audouin’s gulls suggest that transient losses mostly correspond to survival costs of first reproduction^13,29,39,51^. Distinguishing factors such as age and competition on costs of reproduction is important from an evolutionary point of view, because individuals have to decide to reproduce or not as well as the amount of resources devoted to reproduction depending on their age and the future chances to reproduce. These decisions also depend on the availability of resources and their variability in space and time, which are the basis for the existence of trade-offs in nature^52,53^. The importance of age for the costs of reproduction is also expected to vary along the fast-slow continuum in life-histories, because individuals can recruit at different ages (from hours to years depending on the organism’s lifespan) and the decision to start breeding for the first time may compromise future survival.

After the appearance of uni-state models, multi-state models were developed as a more suitable CMR tool to estimate costs of reproduction on future survival of species including fish, amphibians, birds and mammals^54–57^ (Table S4). In these multi-state models, the transition between states (for instance between a pre-breeder state to a breeder sate) is estimated conditional on survival. In the framework of our study, an example would be to assess whether breeding for the first time has an effect on local survival, i.e. individuals breeding for the first time may have a higher mortality or permanent dispersal probability. Nevertheless, some studies have not detected such a cost, especially in long-lived species. In these cases, a prudent strategy (for instance by skipping reproduction) by breeding individuals facing survival-reproduction trade-offs may be invoked^32,40,41^. Additionally, females having higher fertilities may also have higher survival and lower probabilities of skipping breeding, which suggest directional selection for higher fertilitye.g.^58–60^ or can result from variation in individual quality that leads to positive correlations in multiple fitness parameters. When breeding experience can be assessed, a reproductive cost is generally found, mostly for first-time breeders of different ages, as a form of filter selection for more robust individuals within the group of experienced breeders^20,61–63^ (see a complete list of papers in Table S1).

### Caveats concerning treatment of transients in uni-state CMR studies

Transients in uni-state models can be interpreted to estimate costs of reproduction or permanent dispersal only for the first breeding attempt^5^. Other costs, such as skipping breeding (e.g., missing a reproductive season because of low body condition) or the probability of breeding in the future^56^ can be assessed with greater flexibility and reliability by multi-state CMR models and the extension of these models made later in multi-event CMR models^55,64^. Note also that we base much of our interpretation on the assumption that the first reproductive event observed was equal to the first time an individual actually bred. However, it is difficult to gauge how often that assumption can be made in many field studies. Depending on the capture effort and the life history of the species this assumption may sometimes be fairly poor and a lack of transience in the GOF test does not necessarily mean that there are no survival costs of reproduction or permanent dispersal. However, the contrary also holds; for instance, intense field monitoring of burrowing petrels, shearwaters and other birds allows researchers to assume that a bird marked for the first time in a previously occupied burrow is a first-time breeder, because breeding adults are extremely philopatric to the burrow, and the occurrence of transience in CMR analysis corresponds to costs of first breeding^30,31,38,65^.

Another limitation is that for many species, such as fish, small and marine mammals and large carnivores, the Robust Design is the commonest CMR model used^66^; in this model, the presence of transients cannot be assessed because there is no GOF test available, even though the ecology of these species (e.g., open populations for fish and marine mammals, dispersing transient individuals for large carnivores) suggests that transients are likely to occur. When a specific test for detecting transients does not exist, transience can still be assessed through several inference methods (Table S5), the simplest of which is inclusion of a different parameter for the first time step after initial breeding. A lack of power (i.e. small sample sizes) can also prevent a reliable 3.SR test for detecting transience. The other side of the coin is that a non-significant test with large sample size does not necessarily mean that there is no transient loss, but that mortality costs or emigration, if they exist, may be low. In these cases, a way to test whether survival after first breeding differs from later survival is by comparing two nested models. Furthermore, very few papers performed a separate 3.SR GOF test for juveniles and reproductive adults, precluding an assessment of the existence of transients in studies marking several age-classes.

When only newborns are marked and transients appear in the GOF of the CJS model, this likely corresponds to a true lower local survival rate of the first ages before animals reach their adult age. To test for the occurrence of transients following first reproduction in those cases, individual CMR histories can be transformed using the first age of reproduction as the first marking event and then grouping animals depending on their age at first reproduction (e.g., our study case). When neither age nor breeding status (e.g., sexual maturation, sabbatical season) of marked individuals can be assigned and there is a lack of fit for 3.SR tests, it is difficult to assess if transients appear as an age effect or because there is transience after first reproduction (most individuals captured for the first time are young or are first time breeders) or eventually resulting from heterogeneity of individual capture probability^67^. If age at marking is recorded, then a model assessing variability of survival with age can be informative about how age and costs of first breeding interact. For our study case, survival of Audouin’s gull initially increases with age but stabilizes by year 3, the potential age of first breeding; it then remains constant until it shows signs of senescence for older individuals. Survival curves with age can provide a graphical clue for the occurrence of costs of first breeding, when there is a decrease in local survival for the main age of first breeding (see Table S5). The life history of the study species (e.g., fast-slow continuum) is also important to interpret the presence of transients and to disentangle the potential sources for its appearance^35,36^.

Some studies on species with known costs of reproduction (e.g., ungulates) do not detect transience effects. One possible reason is that adults are not necessarily marked during their first reproductive attempt, so the sample is a mixture of first-time and experienced breeders (the later not necessarily paying a cost of reproduction). Another reason is that the study species may show long-term costs of reproduction predicted by senescence theory^68,69^, which cannot be detected by uni-state CMR models and the occurrence of transients. In some other cases transience may represent, at least partly, a permanent response to the first trap-handling, which may depend on the time and type of manipulation but may also be species-specific; although this is mostly untestable, it is realistic for some studies and species^70^.

In general, biological interpretations of transients (as well as of recapture probabilities) have to be cautious because they can result from the combination of several ecological processes (including real transience through the study area). For instance, first time breeding could be used by individuals to provide information on site quality. If it is not a good site (with higher chances for failed reproduction), the individual might decide to disperse, in a process that may not be related to a cost of first reproduction. Finally, in some cases (e.g., some cliff nesting seabirds and crevice-dwelling amphibians) researchers commonly start by catching the most confident individuals^71^, which may also be high-quality animals with high survival; this may result in a higher survival for first-marked animals (i.e. an “anti-transience” effect). The contrary may hold true when using baited traps for capture, because individuals in low body condition may be more prone to be caught and marked, as the condition bias hypothesis predicts^72^.

### Guidelines for dealing with transients

We have noted that the only reliable method to disentangle the biological process causing transients (either permanent dispersal or survival costs after first reproduction or a combination of the two) is to have capture–recapture data at a large spatial scale. Potentially, transients could be caused one of the processes at some ages at first breeding and shifting to the other process as individuals recruit at older ages. When these data are not available, the first recommendation is to follow methodological guidelines recently proposed by Genovart and Pradel^5^ for two additional parametrizations that incorporate a transience effect. With these novel parameterizations, we can directly estimate the “transience probability” considering the behavior of the previously caught individuals in the same sample, and importantly, they allow testing biological hypotheses concerning biological processes affecting this probability. Another issue is to consider the marking protocol since studies marking individuals at different ages (i.e. not when individuals reproduce for the first time) should not find transients, unless there is a detrimental effect of first capture and marking on survival. In combination with these methodological issues, it is crucial to use a priori knowledge of both the natural history of the species and the study system. For instance, the prominence of transients in amphibians may result from the inherent patchiness of pond and wetland environments and the ephemeral nature of some of these patches. Table S5 also provides some tips for detecting costs of reproduction in CMR studies disentangling the causes of transients and their ecological interpretations. Even though the identification of the processes causing transients is challenging, it is important to consider its biological meaning to attain a deeper understanding of the demographic, ecological, and evolutionary processes concerning the study species.

### The consequences of transients on local population dynamics

The potential effects of different life-history traits on population growth rate have been assessed by several authors. For example, Calvo and Horvitz^73^ studied the costs of reproduction in orchids using a Markovian process that was numerically represented as a transition-probability population projection matrix, but the parameters of incurring a cost of reproduction were set theoretically and life histories were simulated. In animals, costs of reproduction may lower population growth rate when some breeders (especially first-time, inexperienced individuals) would die (or disperse) and thus do not contribute further to the local population fitness components^74,75^. For instance, population dynamics of Adélie penguins (*Pygoscelis adeliae*) are influenced by changes in adult survival, and also by the presence of transients, which were found among adult breeders in two different studies^25,76^. These costs of first reproduction are expected to be different depending on age and sex at first breeding, especially for animals with slow life histories, so their impact on population dynamics may also depend on the mating system (i.e. the reproductive investment of each sex allocated in a breeding season) and the lifespan of the organism.

Previous studies have also found that survival costs are greater during severe environmental conditions, including those created by negative density-dependence^8,77,78^. Changes in population density affect the evolution of life history traits, not only by changing selection pressures within local populations but also by changing the distribution of population densities at multiple spatial scales. Despite the negative effect of population density on the occurrence of transients, its consequences on population fluctuations have seldom been demonstrated. Nevertheless, the inclusion of trade-offs in population models is increasing in recent years, e.g. for survival costs of reproduction^79–81^. These studies show the importance of the inclusion of survival-reproduction trade-offs to obtain reliable results from population models, for instance projections of population responses to natural and anthropogenic environmental change, including harvesting.

Particularly for long-lived species, adult mortality caused by the costs of reproduction is expected to have significant impacts on population fluctuations. When transients correspond to permanent dispersers, this may also influence both metapopulation and spatially-structured population dynamics through colonization rates and immigration processes to occupied patches^29,82,83^. This influence can be greater because survival costs of reproduction can vary among populations depending on local environmental conditions and the population age-structure^30,31,84^. This variability can also influence the dynamics of metapopulations at a large spatial scale, not only because it promotes colonization, but also because costs of reproduction in colonized patches during the first phases of population growth may be lower until density-dependence appears^85^.

We showed here that the decrease in λ detected for our study case when the environment became harsher was influenced by the increase in the proportion of transients, even though sensitivities and elasticities of this parameter are much lower than those for adult survival. The potential influence of transients could in fact be even higher than we estimate, because our method cannot detect transience for animals already marked, which may correspond to other costs of reproduction, such as future reproductive success, cost in senescent individuals or the frequency of sabbatical years^17,86–88^. Our life table response experiment shows a large contribution of the occurrence of transients to shifts in λ and hence local population dynamics even though their sensitivity is relatively small. As has been previously pointed out, it cannot be assumed that small effects on the vital rates translate into small contributions to the effects on λ^34,89^. This result has implications for ecological and evolutionary studies and may stimulate other researchers to explore the ecological processes behind the occurrence of transients in capture–recapture studies, as well as the inclusion of these processes to make more reliable population predictions about endangered and harvested species.

In summary, we suggest that ecologists and evolutionary biologists carefully consider the two potential non-exclusive biological explanations for the appearance of transience (survival cost of first breeding and permanent dispersal) and recognize that estimates of transients can be used to make inferences regarding the ecology and life history of their study organisms. In some specific, particular cases, which of these underlying processes accounts for most transience can be inferred, although the validity of that interpretation relative to other possible ecological processes depends strongly on the knowledge of the study system and the life history of the study species e.g.^36,63,90^.

## Conclusion

Many capture–recapture studies on different taxa have found transients. This pattern has been mostly used technically, to make corrections to avoid biased estimates of survival and recruitment, but transients have seldom been considered as a biological process with potential consequences for the population dynamics of the study species. A clue to find out whether transients may correspond to costs of first reproduction is assessing the field protocols to mark and recapture individuals. Using individuals marked or recaptured as first-time breeders ensures that the presence of transients suggests local survival costs of first reproduction. This is especially so when individuals are resighted, i.e. when there is no manipulation that may affect the probability of returning to the study area in successive occasions. While in most cases it is not possible to disentangle whether first- time breeders disappearing from the study areas are either dead or have dispersed permanently, some studies suggest that most of them pay a cost of first reproduction. Capture–recapture models are in these cases a reliable and powerful tool to estimates the survival costs due to lack of experience and assess biological hypothesis related to these costs (e.g. environmental stochasticity). New implementation for a better parameterization of transients have recently been set, which can allow researchers to directly estimate the probability that a newly caught individual disappear from the population^5^. Furthermore, these costs should be included in population models to the study of evolutionary strategies (e.g. life histories) and ecological predictive studies (e.g. harvesting, conservation).

## Materials and methods

### Transients in capture–recapture modeling

Most survival analyses using uni-state models are based on the Cormack-Jolly-Seber (CJS) model, whose assumptions have to be checked through a goodness-of-fit test (GOF)^1,5^. One assumption of these models is that individuals within a given stage or class (e.g., all breeders, all 1 year old, etc.) have the same probability of being recaptured, and in particular that the probability of being recaptured of animals marked for the first time and the probability of being recaptured of animals already marked should be equal^5^. Table S6 shows technical details about what transients mean in the framework of CMR modelling and how they are calculated. This calculation was used for modelling in Table S3. In summary, transients are detected by running a specific goodness-of-fit (GOF) test (namely test 3.SR). To quantify the frequency of transients in CMR studies, we reviewed the literature (Web of Science) using the keywords “transient”, “transience”, “survival” and “capture–recapture” in combination or alone (we note here that “transient” has a different meaning for studies dealing with dynamical systems). We used single searching terms without “transient” and “transience” because many studies did not explicitly mention this potential effect in their methods or results. We also took into account whether a study marked both juveniles and immatures, since detecting transients should correspond to a real decrease of survival for younger animals. We only considered studies performed in breeding areas, with uni-state type data, and discarded studies on wintering survival (mostly on birds) that cannot detect a cost of reproduction. Papers that did not specify which components of the GOF test were statistically significant or did not provide specific results for test 3.SR or similar tests were also not included. The review started with papers published after Pradel et al.^6^, who formally described transients in CMR and how to correct models to avoid biased survival estimates. Papers analyzing the GOF tests by means of RELEASE option in MARK software were accepted with caution because this procedure does not have a directional test to assess the presence of transience, as U-Care software does^91^. Test 3 from RELEASE is the equivalent of tests 3.SR + test 3.Sm given by U-Care. That test helps to detect transients but with lower power than test 3.SR, which was designed specifically for this purpose.

### A case study

Several papers^51,92^ have found a strong transient effect on Audouin’s gull (*Ichthyaetus audouinii*), a long-lived social bird, and we use this species as a case study. In this case, because birds enter the database at different ages (the age at first observation at the colony as a sexually mature bird), transients do not directly correspond to an age effect (i.e. to an increase in survival with age). Two studies working at large spatial scales covering most of the range of the species and including the main breeding sites and 85% of the total world population (i.e. including dispersal among sites to correct the estimation of local survival) showed that transients still appeared, which suggest that an important proportion of transients correspond to individuals dying after first breeding^29,39^.

We updated a CMR database for Audouin’s gull collected over 25 years. During 1988–2012 a total of 24,038 chicks were individually marked at fledging in the Ebro Delta colony using a plastic band with a unique alphanumeric code. We obtained a total of 50,038 resightings on 8423 adult birds over the study. We discarded histories previous to adult (i.e., breeding) age to avoid bias introduced by a lower survival for younger, non-adult birds. Resightings were made during the breeding season (April–July) using spotting scopes. We ran a CMR model in E-SURGE^93^ using a uni-state database on adult gulls to assess whether the proportion of transients was affected by the age at first breeding or by density-dependence, estimated by the time-varying ratio between food availability (measured by the amounts of discarded fish that are largely exploited by opportunistic gulls) and population size of Audouin’s gulls plus that of a key competitor, yellow-legged gulls (*Larus michahellis*), i.e. food-per-capita (see details on density-dependence metrics in^22,94^). Taking into account that recruitment to the breeding class starts at 3 years old and declines sharply for birds older than 6 years of age^22^, age of first breeding was treated with five groups: 3 years, 4 years, 5 years, 6 years and > 6 years old. Table S3 shows the results of the CMR modelling analysis including GOF tests performed for each age-group. All component tests specifically designed to detect transients were highly statistically significant (2275 of the 8423 histories (27%) contained a single mark event). Transients accounted for 95% of the lack of total fit (ĉ = 6.76).

We constructed a life cycle for Audouin’s gull (Fig. 4) that includes transients and incorporates transient effects on survival through τ_i_ survival terms, which reflect the additional mortality above and beyond that experienced by birds of the same age that are not first-time breeders. As emphasized above, two non-exclusive explanations apply for these transients: a survival cost (i.e. mortality) and permanent dispersal. Although at the individual level the fitness consequences for dispersing cannot be estimated, those two explanations had the same consequences for local population dynamics. We parameterized the population matrix model based on the life cycle (Fig. 4) using the mean vital rates estimated from our CMR analyses (for survival and transient probabilities, Table S3). The other vital rates used in the models were fertility and recruitment (see above). We took the values of those vital rates to calculate deterministic population growth rate λ as well as sensitivities and elasticities (Fig. S1). Sensitivities reveal how small changes in a demographic parameter included in the matrix influence population growth rate λ, whereas elasticities estimate the effect of the proportional change in this parameter on λ (see below). To assess the influence of transients and population size on population growth rate, we ran our population model with a range of values for each of these two parameters, keeping the mean values estimated during the study for the rest of vital rates. Analyses were carried out in R^95^ using the library ‘popbio’ available at the CRAN (Comprehensive R Archive Network) repository site. Finally, we measured the consequences at a population level of having transient individuals using a life table response experiment LTRE^96^. LTRE analyses are used to explore changes in population growth rate λ over time or space resulting from experimental manipulations, changes in environmental conditions (e.g., artificial exposure to pollutants, food provisioning, or manipulation of predator densities)^97^, or alternative assumptions about life history or vital rate values. Here, our objective in using LTRE was to assess the impact of transient and other vital rate differences (estimated in the CMR analysis, see above) in generating differences in λ between two contrasting environmental conditions, which in our case are indicated by conditions of low vs high density-dependence seen in years 1993 and 2008 (which we refer to as the ‘good’ and ‘bad’ seasons, respectively). We compared the LTRE contributions of each vital rate with sensitivities and elasticities obtained from our deterministic population model (see above). Following Caswell (2001) for LTRE analysis, we calculated elasticities from the matrix using parameters for the good season, whereas sensitivities came from the mean matrix (i.e. mean values of parameters) between the good and the bad season. Comparing two contrasting seasons with very different environmental conditions is the best procedure to seize LTRE methodology. LTER analyses do not take into account temporal variability in vital rates, but they are still useful when comparing mean vital rates between two scenarios that show contrasting demographic patterns or environmental conditions, such as density-dependence differences in our example^98–100^.

## Supplementary information

Supplementary Information.
