# Supplementary material for: Breeding transients in capture–recapture modeling and their consequences for local population dynamics
Source: Sci Rep. 2020 Sep 25;10:15815. doi: 10.1038/s41598-020-72778-x (PMC7519680; doi:10.1038/s41598-020-72778-x)
Supplement: Supplementary file 1 — Supplementary Information. [file 41598_2020_72778_MOESM1_ESM.pdf]

Supplementary information for article titled:

Breeding transients in capture-recapture modeling and their consequences for local population dynamics

Authors: Daniel Oro, Daniel F. Doak

Supplementary Table S1. Complete table of papers finding transients in uni-state CMR studies.

To assess the proposed basis of transience, when there was an explicit statement about the presence of transients, we examined statements by the authors in the text of their papers. Authors suggested that numerical transients can be the result of age-specific variation in survival or site fidelity, handling effects, or be individuals in passage or floaters [1–3]. Some statements were also made about a potential lower local survival in adults breeding for the first time but it was generally interpreted as age variation in survival without mentioning a potential reproductive cost [4–6]. Only one paper [7] clearly addressed transients as a way to quantify the costs of reproduction (see Table 1 in main text). See references at the end of the Appendix.

**Species**	**Marked individuals**	**Biological process or methodological bias proposed**	**Reference**
Cory's shearwaters	Breeding adults	Cost of first reproduction	[8]
Sooty shearwaters	Adults, juvenile and pre-breeding birds	Prospecting	[9]
Long-tailed Jaegers	Adults	True age effect (if a high proportion of young adults was present in the initial samples) or more likely, presence of transient individuals (maybe prospectors)	[10]
Dunlin	First-years and adults	Capture and release may induce short-term behavioural changes that increase the risk of predation by avian predators within the first few days after capture; could be also actual transients	[11]
Black-capped chickadee	Unknown age	Age-effect, maybe also some actual transients	[12]
Balearic shearwater<br>Storm petrel	Breeding adults<br>Breeding adults	Cost of first reproduction (but just in one of the two study colonies)<br>Cost of first reproduction and/or a predatory effect (but just in one of the two study colonies)	[13]

Species	Marked individuals	Biological process or methodological bias proposed	Reference
Alpine Newt	Adults	Nomadic behaviour that contribute to breeding dispersal; different between local populations	[14]
Toad (*Bufo bufo*)	Unknown age	True age effect	[15]
Swainson's thrush	Adults, juveniles	True age effect	[16]
Tiger	Adults	Transience over the study area	[17]
Grey seal	Adults	Not interpreted, but authors stated that "animals seen for the first time in any year (i.e., first time breeders) are somewhat less likely to be seen in subsequent years", and this suggests costs of first reproduction	[5]
Polar bears	Adults	Strong Test 3.SR for males, slight 3.SR for females; no explanation	[18]
Common eiders	Breeding females	Test 3.SR significant; no explanation, but it seems a cost of reproduction	[19]
17 species of tropical passerines	Unknown age	Netting-trapped individuals; recently fledged birds are likely to have been common in mist-net samples. Found transients in 65% of the species, but probably was an age effect	[20]
Barn swallows	Breeding adults	Test 3.SR significant for females only in some years, but not for males. No clear explanation is given.	[21]
Greater flamingos	Chicks	A cost of reproduction for first-time breeding females	[7]
Green turtles	Immatures	Real transients (or an age effect) in one population but not in the other	[22]
Least and Cassin's Auklets	Non-breeding and breeding adults	Transients assumed to be non-breeding prospectors	[23,24]
Emperor penguins	Breeding adults	Models with 2 age-classes, no explanations for this	[25]
Pipistrelle bats	Juveniles and adults	Transients in autumn, when some bats disperse permanently	[3]
Loggerhead turtle	Juveniles and adults	Transients only among juveniles: age effect or transients	[26]
Wood mice	Juveniles and	Transients detected and not interpreted, but corrected with $\hat{c}$	[27]

Species	Marked individuals	Biological process or methodological bias proposed	Reference
Bank voles	adults<br>Juveniles and adults	Transients detected and not interpreted, but corrected with ĉ	
Florida manatee	Adults	Lack of fit in 3 of 4 of study populations, corrected with ĉ	[28]
Black-footed albatross	Adults	They treated the lack of fit of GOF test using a temporary age-dependent emigration model; no costs of reproduction were considered	[29]
Green sea turtle	Adults, sub-adults and juveniles	Found a transient effect for males and females sub-adults and for juveniles (not for adults): authors considered that there was a real transient effect for the two first age-classes	[26]
Snow petrel	Adults	Lack of fit corrected with ĉ, no biological interpretation	[30]
Blue petrel	Adults	Lack of fit corrected with ĉ, no biological interpretation	[31]
Southern fulmar	Adults	Lack of fit corrected with ĉ, no biological interpretation	[32]
New Zealand long-tailed bats	All ages	Lack of fit corrected with ĉ, no clear biological interpretation	[33]
Fire salamanders	Adults	Assumed that was caused by permanent emigration	[34]
Common guillemots	Breeding adults	Strong transient effect in 2 of the 3 study colonies, corrected with ĉ, no clear biological interpretation	[35]
Roseate terns	Breeding adults	Assumed transients emigrate to other colony sites after first breeding attempt	[36]
Field vole	Juveniles and adults	Assumed an age effect; the first captures of all individuals were removed in order to overcome biases caused by individuals only caught in a single primary session (transients)	[37]
Bottlenose dolphins	Sub-adults and adults	Transients especially for adults, but also for sub-adults; no explanation given	[38]
Piping plovers	Juveniles and adults	Transients among adults only for one specific occasion; no explanation	[39]

Species	Marked individuals	Biological process or methodological bias proposed	Reference
Ruby-throated hummingbirds	Adults separately from juveniles	Transients higher for males; authors assumed that males dispersed more than females and they left the study area	[40]
Cerambyx welensii (beetle)	Adults	Transients especially for males in most years; authors assumed they corresponded to permanent emigration, maybe following disturbances	[41]
Common toads	Adults	No explanation	[42]
American redstart<br>Ovenbird	Adults<br>Adults	GOF not performed; authors state that transients might be non-territorial floaters or failed breeders<br>Idem	[43]
Cory's shearwater	Adults	Transients only in 1 of the 6 study colonies; no explanation	[44]
Grey mouse lemur	Juveniles and adults	Juvenile males disperse; no transients for adults and juvenile females	[45]
Audouin's gull	Adults	Permanent dispersal	[46,47]
Adélie penguin	Juveniles and adults	Adult breeding males showed transience particularly in one year; assumed that it was a bad year triggering permanent dispersal	[48]
Swainson's Thrushes	Adults	Floaters and migratory individuals	[49]
Leatherback sea turtle	Female adults	Permanent dispersal	[50]
Northern spectacled salamander	First breeding females	Transients occurred at one of the two study sites; authors suggested permanent emigration caused by density-dependence (see also Perret et al. 2003)	[51]
Common toad	Breeding adults	Transients only for males; no clear explanation, authors suggested permanent dispersal or an age effect on survival	[52]
Apron (*Zingel asper*)	Adults	Transients occurred at the three study sites; assumed it was permanent dispersal to sub-optimal sites or fish were highly mobile	[53]
Black Guillemot	Breeding adults	An age effect	[54]
Cottonmouth	Adults and	No explanation	[55]

Species	Marked individuals	Biological process or methodological bias proposed	Reference
	juveniles		
Kentish plover	Juveniles and adult breeders	Several explanations but none related to reproductive costs	[2]
Semipalmated plover	breeders	Several explanations but none related to reproductive costs	

Supplementary Table S2. Specification of the multi-event modeling approach in program E-SURGE

We treated a uni-state modeling by using a multi-event approach. A multi-event model was built in several stages using program E-SURGE. Each step represents the different parameters to estimate. This is done by means of row-stochastic matrices, i.e. each row corresponds to a multinomial distribution. Consequently, the total of cell probabilities is 1. Because of this constraint, one and only one cell probability in each row will be calculated as the complement to 1 of the others. This particular cell is denoted with a '*' symbol. Inactive cells, i.e. cells whose associated probability is structurally 0 are denoted with a '-' symbol. An active cell receives an arbitrary letter. Find below details on the multi-event modeling analysis carried out in this study.

The individual states considered are:
AA: alive and aware
AU: alive and unaware
D: dead

The possible events are:
0: not recaptured
1: recaptured alive

The symbols for parameters are:
ϕ: Survival probability

We considered two age classes (a). These two ages all relate to adult individuals but represent those encountered for the first time, and those encountered more than once. In E-SURGE, the pattern matrices were:

Initial State probabilities

AA	***AU***
*	-

Transition probabilities, step 1: Survival

	AA	***AU***	***D***
AA	ϕ	-	*
AU	-	ϕ	*
D	-	-	*

Transition probabilities, step 2: Recapture

	AA	***AU***	***D***
AA	***p***	*	-
AU	***p***	*	-
D	-	-	*

Event probabilities: observation process

	0	***1***
AA	-	*
AU	*	-
D	*	-

The GEMACO definition for best model in Table S3 (model 1 below in Table S3) was:

Initial step 1:
- i

Transitions
- Transition step 1 (Survival):
  [a(1).g.t]+[a(2:25).t]
- Transition step 2 (Recapture):
  f.t

The GEMACO definition for density-dependence model in Table S3 (model 6 below in Table S3) was:

Initial step 1:
- i

Transitions
- Transition step 1 (Survival):
  [a(1).g(1,3,4,5).t]+ [a(1).g(2).[t*x(1)]]+[a(2:25).t]
  x=(-2.2567, -0.90189, -0.50958, -0.41583, 0.094181, 0.29645, 0.27913, -0.48614,-0.30883, 0.26639, -0.52028, -0.40156, -1.0063, 1.4495, 2.1327, 1.5458, 0.96186, -0.7037, 0.06514, 0.41975)
- Transition step 2 (Recapture):
  f.t

Supplementary Table S3. Capture-mark-recapture CMR modelling analysis on Audouin's gull from the Ebro Delta colony during 1988-2012 and development of the projection matrix used for population modelling in R (see Methods for more details)

For the Audouin's gull study, a goodness of fit test (GOF) indicated a large lack of fit of the starting general Cormack-Jolly-Seber model CJS (global TEST, number of groups =12, df = 585, $\chi_2^2 = 1707.4606$, p < 0.0001); this was caused by the presence of both trap-dependence and transience effects (N(0,1) signed statistic for trap-dependence =-18.3849, P-level, two-sided test =0; N(0,1) statistic for transient(>0) =12.8401, P-level, two-sided test =0). Since we only took into account the capture-recapture histories starting with the age of first reproduction (i.e. adult birds) transience was not likely caused by an age effect (survival for this species has already stabilized before reaching adult ages), but probably results from a cost of first reproduction [56,57]. To deal with the presence of transients and trap-dependence in the CJS model, we built CMR models under the multi-event framework using E-SURGE. Multi-event models relate the true state of the individual (i.e. present at the colony – dead) with the observed event (i.e. seen – not seen) through a series of conditional probabilities [58,59]. Estimation of the proportion of transients from the survival of transients and resident individuals is shown in Table S6.

The following table shows the results of the modeling of survival of breeding Audouin's gull at the Ebro Delta during 1988-2012: # Par is the number of identifiable parameters; Dev = deviance of the model *i*; QAIC = AIC value corrected for overdispersion; ΔQAIC = difference in QAIC value with the best model; w_i = weight of model *i*. Models are ordered by QAIC value. Model notation: τ = transients; ϕ = survival; a = age; p = resightings probability; t = time; · = constant; DDT = density-dependence (in terms of amount of trawling discards available) for the total population of Audouin's and yellow-legged gulls; DDLa = density-dependence only for Audouin's gulls; T =temporal trend. Only the best models are shown.

	Model	Hypothesis for transients	Hypothesis for survival	# Par	Dev_i	QAIC	ΔQAIC	w_i
1	$\tau_{a.t}\ \phi_t\ p_t$	Changed with age and time	Changed with time	150	67706.81	68008.27	0	1
2	$\tau_{a.t}\ \phi_a\ p_t$		Changed with age	152	67745.41	68050.91	42.64	0.00
3	$\tau_{a.t}\ \phi_{a.t}\ p_t$		Changed with age and time	321	67422.41	68071.09	62.82	0.00
4	$\tau_{a+t}\ \phi_t\ p_t$	Changed with time and additively for each age	Changed with time	82	67915.54	68079.98	71.71	0.00
5	$\tau_{a+T}\ \phi_t\ p_t$	Changed with an increasing temporal trend and additively for each age		64	67956.74	68085.01	76.74	0.00
6	$\tau_{a+DDT}\ \phi_t\ p_t$	Changed with age and an increasing trend with total density-dependence		64	67972.38	68100.65	92.38	0.00
7	$\tau_{a+DDLa}\ \phi_t\ p_t$	Changed with age and an increasing trend with density-dependence for Audouin's gulls		64	67999.71	68127.98	119.71	0.00
8	$\tau_{a.t}\ \phi_\cdot\ p_t$	Changed with age and time	Constant	134	67864.50	68133.66	125.40	0.00
9	$\tau_a\ \phi_t\ p_t$	Changed with age	Changed with time	63	68043.25	68169.51	161.24	0.00
10	$\tau_{a.t+DDT.4y}\ \phi_t\ p_t$	Changed with age and an increasing trend with total density-dependence only for the modal age of first breeding (4y old birds)		133	68407.86	68675.01	666.74	0.00
11	$\tau_{a.t+DDLa.4y}\ \phi_t\ p_t$	Changed with age and an increasing trend with density-dependence for Audouin's gulls only for the modal age of first breeding (4y old birds)		133	68415.50	68682.65	674.38	0.00
12	$\tau_{a.T}\ \phi_t\ p_t$	Changed with age and a trend with time		63	71124.52	71250.78	3242.51	0
13	$\tau_{a.t}\ \phi_T\ p_t$	Changed with age and time	A trend with time	134	85049.61	85318.77	17310.50	0

The best model included an interaction of age and time on transient probabilities (model 1 in the table above), but in our subsequent matrix analyses in which we use transient parameters we used the additive model (model 4) for simplicity We note that this model was much better than the one with only either age or time. We use the ANODEV procedure to estimate the effect of total density-dependence (DDT, model 6) on transient probabilities [60].

Supplementary Figure S1. Representation of the pre-breeding census population projection matrix used for population modelling. In this matrix, classes 1-6 are pre-breeding birds, while classes 7-11 represent first time breeders. Class 13 includes all birds surviving after first breeding and class 12 are transients. Note that since transients are not seen again, this class can be eliminated from the transition matrix for estimating population growth but we include it here to correspond as closely as possible to Figure 3 in the main text.

N_1	(0,	0,	0,	0,	0,	0,	$x*F'*\phi_1$,	$x*F'*\phi_1$,	$x*F'*\phi_1$,	$x*F'*\phi_1$,	$x*F'*\phi_1$,	0,	$x*F*\phi_1$,
N_2	ϕ_2,	0,	0,	0,	0,	0,	0,	0,	0,	0,	0,	0,	0,
N_{INB3}	0,	$\phi_A*(1-\gamma_3)$,	0,	0,	0,	0,	0,	0,	0,	0,	0,	0,	0,
N_{INB4}	0,	0,	$\phi_A*(1-\gamma_4)$,	0,	0,	0,	0,	0,	0,	0,	0,	0,	0,
N_{INB5}	0,	0,	0,	$\phi_A*(1-\gamma_5)$,	0,	0,	0,	0,	0,	0,	0,	0,	0,
N_{INB6}	0,	0,	0,	0,	$\phi_A*(1-\gamma_6)$,	0,	0,	0,	0,	0,	0,	0,	0,
N_{IB3}	0,	$\phi_A*\gamma_3$,	0,	0,	0,	0,	0,	0,	0,	0,	0,	0,	0,
N_{IB4}	0,	0,	$\phi_A*\gamma_4$,	0,	0,	0,	0,	0,	0,	0,	0,	0,	0,
N_{IB5}	0,	0,	0,	$\phi_A*\gamma_5$,	0,	0,	0,	0,	0,	0,	0,	0,	0,
N_{IB6}	0,	0,	0,	0,	$\phi_A*\gamma_6$,	0,	0,	0,	0,	0,	0,	0,	0,
N_{IB7}	0,	0,	0,	0,	0,	$\phi_A*\gamma_7$,	0,	0,	0,	0,	0,	0,	0,
N_{exp}	0,	0,	0,	0,	0,	0,	$\phi_A*\tau_3$,	$\phi_A*\tau_4$,	$\phi_A*\tau_5$,	$\phi_A*\tau_6$,	$\phi_A*\tau_7$,	0,	0,
N_{it}	0,	0,	0,	0,	0,	0,	$\phi_A*(1-\tau_3)$,	$\phi_A*(1-\tau_4)$,	$\phi_A*(1-\tau_5)$,	$\phi_A*(1-\tau_6)$,	$\phi_A*(1-\tau_7)$,	0,	ϕ_A)

In which:
x: sex ratio
ϕ_1: juvenile survival
ϕ_2: immature survival
ϕ_A: adult survival
γ_i: recruitment for age class *i*
τ_i: transients for age class *i*
F': fertility for first-time breeders
F: fertility for “experienced” breeders

Supplementary Table S4. Results of a literature search for papers dealing with the costs of reproduction using multi-state CMR modelling. Some papers are cited more than once because multiple drivers and factors were found. See references at the end of the Appendix.

Species	Drivers and factors involved	References
Cory's shearwater, yellow-bellied toad, brown trout	Harsh conditions during previous winter may decrease survival or future breeding after reproduction	[8,61,62]
Kittiwake, tiger salamander	Reduced food availability during breeding increase survival cost of reproduction	[63,64]
yellow-bellied toad	Future reproduction costs higher for females	[61]
Soay sheep, great tit	Survival reproductive costs of senescence	[65,66]
Soay sheep, Alpine ibex	Survival costs only appear during disease outbreaks and during severe environmental conditions, including density-dependence	[65,67]
Kittiwake, common eider, blue petrel, fur seal, Nazca booby, reindeer, sage-grouse, red deer	Costs differ depending on the individual quality of females	[19,68–74]
blue petrel, Soay sheep, fur seal, Weddell seal, storm petrel, elephant seal	Costs (either survival or subsequent reproductive probabilities) are higher for inexperienced breeders	[65,69,70,75–77]
Field vole	Survival costs of reproduction can vary between and within populations and be	[78]

	particularly large in declining populations	

Supplementary Table S5. **Some tips for detecting costs of reproduction in CMR studies disentangling the causes of transients and their ecological interpretations**

- When newborns are marked, a transient effect also appears in the GOF of the CJS model, but this corresponds to a true lower survival of the first age. Some papers tested the GOF separating newborns from the rest of age classes to take this into account and test for a potential cost of reproduction for animals reproducing for the first time
- In several cases, a study included a mixture of marked resident and transient individuals. For example Nur [79] suggested the use of multiple captures in a single recapture occasion to distinguish the two type of breeders in passerines caught in nets during the reproductive season.
- When a transient effect on reproducing adults is strong and statistically significant, then it is clear that some significant underlying biological process is occurring (e.g. permanent dispersal, cost of reproduction). But even when the test is not significant there may be some of these processes acting, though not strongly enough to be detected (and corrected). So the lack of statistical significance does not mean that such processes do not occur, but simply that they are likely weak relative to the sampling effort. For instance, [80] working on polar bears stated that "The female data fit model ϕ_t,p_t marginally well (overall $\chi 2_{125}$ = 150.19, *P* = 0.06)", so it seems that real transients and a cost of reproduction might occur, but they are not extremely strong. "Prime-adults" of male polar bears (aged between 5-19 years, so sexually mature males) showed a stronger transient effect (once again authors did not give a biological explanation, though they rightly correct for this effect in survival estimation).
- Methodologically, if juveniles and adults are marked, a GOF should be carried out after removing the first capture, as well as a GOF for a model only including individuals marked as adults to check for potential transients among adults. But this only works if adults appear in the population as breeders.
- Survival curves with age can also provide a graphical clue for the occurrence of costs of first breeding, when there is a decrease in local survival for the main age of first breeding. For instance, the figure below shows the survival curves with age (mean and 95%CI) for two bird species, Adélie penguin (solid line) and Egyptian vulture (dashed line); the commonest age at first breeding is shown with white dots (4 and 5y old respectively). Adapted from [48,81] respectively.

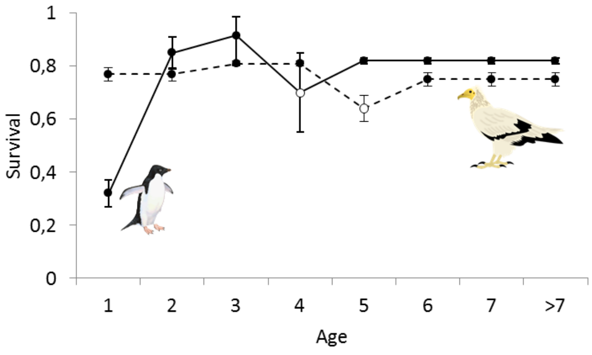

Supplementary Table S6. Technical details about transients in the framework of CMR modelling

Most of the survival analysis using uni-state models are based on the Cormack-Jolly-Seber (CJS) model, whose assumptions have to be checked through a goodness-of-fit test (GOF)[82]. One of the CJS model assumptions is that individuals within a given stage or class (e.g., all breeders, all one year olds, etc.) have the same probability of being recaptured, and in particular to that the probability of being recaptured of animals marked for the first time and the probability of being recaptured of animals already marked should be equal. In a simple tabular form this assumption is that $f1 \approx f3$ and $f2 \approx f4$ in:

Recaptured before	Recaptured again	Not recaptured again
No	*f1*	*f2 +++*
Yes	*f3*	*f4*

where *fi* refers to the frequency of individuals marked and recaptured in each category. When a χ^2 applied to this contingency table is highly significant (very often because *f2* is much larger than expected) then the CJS is not valid as a starting model because there is a so-called "transient" effect [83]. This test is called 3.SR in CMR uni-state modelling and it is calculated using program U-Care [84]. In this case, a starting model with a different (i.e. lower) survival for the first-time marked animals is needed, since otherwise biological conclusions can be wrong. For instance, Brawn et al. (1998)

noted that the lack of correction for the occurrence of transients has frequently resulted in biased estimates of the survival rates of tropical birds. Transients can also be detected with other techniques (such as radio-tracking individuals) to obtaining less biased survival estimates.

Technically it is easy to calculate the proportion of transient individuals of a given age or stage *i*, τ_i, as the complementary probability of the ratio between survival of transients ϕ' over survival of resident individuals ϕ:

$$\tau_i = 1 - \frac{\phi_i^{'}}{\phi_i}$$

Using these age or stage values of τ, the proportion of transients can be calculated for the entire population. The estimation of the variance of τ (σ_τ) is less straightforward and requires the use of delta method and variance-covariance matrices.

References

1. Nott, M. P. & DeSante, D. F. Demographic monitoring and the identification of transients in mark-recapture models. in *Predicting species occurrences: issues of accuracy and scale* (eds. Scott, J. et al.) 727–736 (Island Press, 2002).
2. Sandercock, B. K. Estimation of survival rates for wader populations: a review of mark-recapture methods. *Wader Study Group Bull.* **100**, 163–174 (2003).
3. Sendor, T. & Simon, M. Population dynamics of the pipistrelle bat: effects of sex, age and winter weather on seasonal survival. *J. Anim. Ecol.* **72**, 308–320 (2003).
4. Sandercock, B. K. & Gratto-Trevor, C. L. Local survival in Semipalmated Sandpipers Calidris pusilla breeding at La Pérouse Bay, Canada. *Ibis* **139**, 305–312 (1997).
5. Schwarz, C. J. & Stobo, W. T. Estimation of juvenile survival, adult survival, and age-specific pupping probabilities for the female grey seal (Halichoerus gryprus) on

Sable Island from capture-recapture data. *Can.J.Fish.Aquat.Sci.* **57**, 247–253 (2000).

6. Blake, J. G. & Loiselle, B. A. Estimates of Apparent Survival Rates for Forest Birds in Eastern Ecuador. *Biotropica* **40**, 485–493 (2008).
7. Tavecchia, G., Pradel, R., Boy, V., Johnson, A. R. & Cézilly, F. Sex- and age-related variation in survival and cost of first reproduction in greater flamingos. *Ecology* **82**, 165–174 (2001).
8. Genovart, M. *et al.* Contrasting effects of climatic variability on the demography of a trans-equatorial migratory seabird. *J. Anim. Ecol.* **82**, 121–130 (2013).
9. Clucas, R. J., Fletcher, D. J. & Moller, H. Estimates of adult survival rate for three colonies of Sooty Shearwater (Puffinus griseus) in New Zealand. *Emu* **108**, 237–250 (2008).
10. Julien, J. R., Gauthier, G., Morrison, R. I. G. & Bêty, J. Survival Rate of the Long-Tailed Jaeger at Alert, Ellesmere Island, Nunavut. *The Condor* **115**, 543–550 (2013).
11. Warnock, N., Page, G. W. & Sandercock, B. K. Local survival of Dunlin wintering in California. *Condor* **99**, 906–915 (1997).
12. Loery, G., Nichols, J. D. & Hines, J. E. Capture-recapture analysis of a wintering black-capped chickadee population in Connecticut, 1958-1993. *Auk* **114**, 431–442 (1997).
13. Tavecchia, G., Minguez, E., De León, A., Louzao, M. & Oro, D. Living close, doing differently: Small-scale asynchrony in demography of two species of seabirds. *Ecology* **89**, 77–85 (2008).
14. Perret, N., Pradel, R., Miaud, C., Grolet, O. & Joly, P. Transience, dispersal and survival rates in newt patchy populations. *J. Anim. Ecol.* **72**, 567–575 (2003).

15. Schmidt, B. R., Schaub, M. & Anholt, B. R. Why you should use capture-recapture methods when estimating survival and breeding probabilities: on bias, temporary emigration, overdispersion, and common toads. *Amphib.-Reptil.* **23**, 375–388 (2002).

16. Gardali, T., Barton, D. C., White, J. D., Geupel, G. R. & Thompson III, F. R. Juvenile and adult survival of swainson's thrush (catharus ustulatus) in coastal california: annual estimates using capture-recapture analyses. *The Auk* **120**, 1188–1194 (2003).

17. Karanth, K. U., Nichols, J. D., Kumar, N. S. & Hines, J. E. Assessing tiger population dynamics using photographic capture–recapture sampling. *Ecology* **87**, 2925–2937 (2006).

18. Regehr, E. V., Lunn, N. J., Amstrup, S. C. & Stirling, I. Effects of Earlier Sea Ice Breakup on Survival and Population Size of Polar Bears in Western Hudson Bay. *J. Wildl. Manag.* **71**, 2673–2683 (2007).

19. Yoccoz, N. G., Erikstad, K. E., Bustnes, J. O., Hanssen, S. A. & Tveraa, T. Costs of reproduction in common eiders (Somateria mollissima): An assessment of relationships between reproductive effort and future survival and reproduction based on observational and experimental studies. *J. Appl. Stat.* **29**, 57–64 (2002).

20. Johnston, J. P., Peach, W. J., Gregory, R. D. & White, S. A. Survival Rates of Tropical and Temperate Passerines: A Trinidadian Perspective. *Am. Nat.* **150**, 771–789 (1997).

21. Møller, A. P. & Szép, T. Survival rate of adult barn swallows hirundo rustica in relation to sexual selection and reproduction. *Ecology* **83**, 2220–2228 (2002).

22. Bjorndal, K. A., Bolten, A. B. & Chaloupka, M. Y. Survival probability estimates for immature green turtles Chelonia mydas in the Bahamas. *Mar. Ecol. Prog. Ser.* **252**, 273–281 (2003).
23. Jones, I. L., Hunter, F. M. & Robertson, G. J. Annual adult survival of Least Auklets (Aves, Alcidae) varies with large-scale climatic conditions of the North Pacific Ocean. *Oecologia* **133**, 38–44 (2002).
24. Bertram, D. F., Harfenist, A. & Smith, B. D. Ocean climate and El Nino impacts on survival of Cassin's Auklets from upwelling and downwelling domains of British Columbia. *Can. J. Fish. Aquat. Sci.* **62**, 2841–2853 (2005).
25. Barbraud, C. & Weimerskirch, H. Emperor penguins and climate change. *Nature* **411**, 183–186 (2001).
26. Chaloupka, M. & Limpus, C. Survival probability estimates for the endangered loggerhead sea turtle resident in southern Great Barrier Reef waters. *Mar. Biol.* **140**, 267–277 (2002).
27. Telfer, S. *et al.* The effects of cowpox virus on survival in natural rodent populations: increases and decreases. *J. Anim. Ecol.* **71**, 558–568 (2002).
28. Langtimm, C. A. *et al.* Survival Estimates for Florida Manatees from the Photo-Identification of Individuals. *Mar. Mammal Sci.* **20**, 438–463 (2004).
29. Véran, S. *et al.* Quantifying the impact of longline fisheries on adult survival in the black-footed albatross. *J. Appl. Ecol.* **44**, 942–952 (2007).
30. Barbraud, C., Weimerskirch, H., Guinet, C. & Jouventin, P. Effect of sea-ice extent on adult survival of an Antarctic top predator: the snow petrel Pagodroma nivea. *Oecologia* **125**, 483–488 (2000).

31. Barbraud, C. & Weimerskirch, H. Climate and density shape population dynamics of a marine top predator. *Proc. R. Soc. Lond. B Biol. Sci.* **270**, 2111–2116 (2003).

32. Jenouvrier, S., Barbraud, C. & Weimerskirch, H. Effects of climate variability on the temporal population dynamics of southern fulmars. *J. Anim. Ecol.* **72**, 576–587 (2003).

33. Pryde, M. A., O'Donnell, C. F. J. & Barker, R. J. Factors influencing survival and long-term population viability of New Zealand long-tailed bats (Chalinolobus tuberculatus): Implications for conservation. *Biol. Conserv.* **126**, 175–185 (2005).

34. Schmidt, B. R., Schaub, M. & Steinfartz, S. Apparent survival of the salamander Salamandra salamandra is low because of high migratory activity. *Front. Zool.* **4**, 19 (2007).

35. Harris, M. P., Wanless, S., Rothery, P., Swann, R. L. & Jardine, D. Survival of adult Common Guillemots Uria aalge at three Scottish colonies. *Bird Study* **47**, 1–7 (2000).

36. Spendelow, J. A., Nichols, J. D., Hines, J. E., Lebreton, J.-D. & Pradel, R. Modelling postfledging survival and age-specific breeding probabilities in species with delayed maturity: A case study of Roseate Terns at Falkner Island, Connecticut. *J. Appl. Stat.* **29**, 385–405 (2002).

37. Burthe, S. *et al.* Cowpox virus infection in natural field vole Microtus agrestis populations: significant negative impacts on survival. *J. Anim. Ecol.* **77**, 110–119 (2008).

38. Silva, M., Magalhães, S., Prieto, R., Santos, R. & Hammond, P. Estimating survival and abundance in a bottlenose dolphin population taking into account transience and temporary emigration. *Mar. Ecol. Prog. Ser.* **392**, 263–276 (2009).

39. Larson, M. A., Ryan, M. R. & Root, B. G. Piping plover survival in the great plains: an updated analysis. *J. Field Ornithol.* **71**, 721–729 (2000).

40. Hilton, B. & Miller, M. W. Annual survival and recruitment in a ruby-throated hummingbird population, excluding the effect of transient individuals. *The Condor* **105**, 54–62 (2003).

41. López-Pantoja, G., Nevado, L. & Sánchez-Osorio, I. Mark-recapture estimates of the survival and recapture rates of Cerambyx welensii Küster (Coleoptera cerambycidae) in a cork oak dehesa in Huelva (Spain). *Open Life Sci.* **3**, 431–441 (2008).

42. Reading, C. J. Linking global warming to amphibian declines through its effects on female body condition and survivorship. *Oecologia* **151**, 125–131 (2006).

43. Bayne, E. M. & Hobson, K. A. Annual survival of adult American redstarts and ovenbirds in the southern boreal forest. *Wilson Bull.* **114**, 358–367 (2002).

44. Jenouvrier, S. *et al.* Global climate patterns explain range-wide synchronicity in survival of a migratory seabird. *Glob. Change Biol.* **15**, 268–279 (2009).

45. Kraus, C., Eberle, M. & Kappeler, P. M. The costs of risky male behaviour: sex differences in seasonal survival in a small sexually monomorphic primate. *Proc. R. Soc. B Biol. Sci.* **275**, 1635–1644 (2008).

46. Oro, D., Pradel, R. & Lebreton, J.-D. Food Availability and Nest Predation Influence Life History Traits in Audouin's Gull, Larus audouinii. *Oecologia* **118**, 438–445 (1999).

47. Oro, D., Cam, E., Pradel, R. & Martinez-Abrain, A. Influence of food availability on demography and local population dynamics in a long-lived seabird. *Proc. R. Soc. B Biol. Sci.* **271**, 387–396 (2004).

48. Ballerini, T., Tavecchia, G., Olmastroni, S., Pezzo, F. & Focardi, S. Nonlinear effects of winter sea ice on the survival probabilities of Adélie penguins. *Oecologia* **161**, 253–265 (2009).

49. Rosenberg, D. K., DeSante, D. F., McKelvey, K. S. & Hines, J. E. Monitoring survival rates of Swainson's Thrush Catharus ustulatus at multiple spatial scales. *Bird Study* **46 (suppl.)**, 198–208 (1999).

50. Rivalan, P. *et al.* Trade-off between current reproductive effort and delay to next reproduction in the leatherback sea turtle. *Oecologia* **145**, 564–574 (2005).

51. Angelini, C., Antonelli, D. & Utzeri, C. Capture-mark-recapture analysis reveals survival correlates in Salamandrina perspicillata (Savi, 1821). *Amphib.-Reptil.* **31**, 21–26 (2010).

52. Frétey, T., Cam, E., Garff, B. L. & Monnat, J.-Y. Adult survival and temporary emigration in the common toad. *Can. J. Zool.* **82**, 859–872 (2004).

53. Labonne, J. & Gaudin, P. Exploring Population Dynamics Patterns in a Rare Fish, Zingel asper, through Capture-Mark-Recapture Methods. *Conserv. Biol.* **19**, 463–472 (2005).

54. Frederiksen, M. & Petersen, A. Adult survival of the Black Guillemot in Iceland. *Condor* **101**, 589–597 (1999).

55. Koons, D. N., Birkhead, R. D., Boback, S. M., Williams, M. I. & Greene, M. P. The effect of body size on cottonmouth (Agkistrodon piscivorus) survival, recapture probability, and behavior in an Alabama swamp. *Herpetol. Conserv. Biol.* **4**, 221–235 (2009).

56. Tavecchia, G., Pradel, R., Genovart, M. & Oro, D. Density-dependent parameters and demographic equilibrium in open population. *Oikos* **116**, 1481–1492 (2007).

57. Fernández-Chacón, A. *et al.* When to stay, when to disperse and where to go: survival and dispersal patterns in a spatially structured seabird population. *Ecography* no–no (2013) doi:10.1111/j.1600-0587.2013.00246.x.

58. Pradel, R. Multievent: An Extension of Multistate Capture–Recapture Models to Uncertain States. *Biometrics* **61**, 442–447 (2005).

59. Genovart, M. & Pradel, R. Transience effect in capture-recapture studies: The importance of its biological meaning. *PLOS ONE* **14**, e0222241 (2019).

60. Grosbois, V. *et al.* Assessing the impact of climate variation on survival in vertebrate populations. *Biol. Rev.* **83**, 357–399 (2008).

61. Cayuela, H. *et al.* To breed or not to breed: past reproductive status and environmental cues drive current breeding decisions in a long-lived amphibian. *Oecologia* **176**, 107–116 (2014).

62. Fernández-Chacón, A. *et al.* Neighbouring populations, opposite dynamics: influence of body size and environmental variation on the demography of stream-resident brown trout (Salmo trutta). *Oecologia* **178**, 379–389 (2015).

63. Oro, D. & Furness, R. W. Influences of food availability and predation on survival of kittiwakes. *Ecology* **83**, 2516–2528 (2002).

64. Church, D. R., Bailey, L. L., Wilbur, H. M., Kendall, W. L. & Hines, J. E. Iteroparity in the variable environment of the salamander Ambystoma tigrinum. *Ecology* **88**, 891–903 (2007).

65. Tavecchia, G. *et al.* Predictors of reproductive cost in female Soay sheep. *J. Anim. Ecol.* **74**, 201–213 (2005).

66. Bouwhuis, S., Choquet, R., Sheldon, Ben C. & Verhulst, S. The Forms and Fitness Cost of Senescence: Age-Specific Recapture, Survival, Reproduction, and Reproductive Value in a Wild Bird Population. *Am. Nat.* **179**, E15–E27 (2012).

67. Garnier, A., Gaillard, J.-M., Gauthier, D. & Besnard, A. What shapes fitness costs of reproduction in long-lived iteroparous species? A case study on the Alpine ibex. *Ecology* (2015) doi:10.1890/15-0014.1.

68. Cam, E., Hines, J. E., Monnat, J. Y., Nichols, J. D. & Danchin, E. Are adult nonbreeders prudent parents? The kittiwake model. *Ecology* **79**, 2917–2930 (1998).

69. Barbraud, C. & Weimerskirch, H. Environmental conditions and breeding experience affect costs of reproduction in blue petrels. *Ecology* **86**, 682–692 (2005).

70. Beauplet, G., Barbraud, C., Dabin, W., Küssener, C. & Guinet, C. Age-specific survival and reproductive performances in fur seals: evidence of senescence and individual quality. *Oikos* **112**, 430–441 (2006).

71. Townsend, H. M. & Anderson, D. J. Assessment of Costs of Reproduction in a Pelagic Seabird Using Multistate Mark–Recapture Models. *Evolution* **61**, 1956–1968 (2007).

72. Weladji, R. B. *et al.* Heterogeneity in individual quality overrides costs of reproduction in female reindeer. *Oecologia* **156**, 237–247 (2008).

73. Moyes, K. *et al.* Individual differences in reproductive costs examined using multi-state methods. *J. Anim. Ecol.* **80**, 456–465 (2011).

74. Blomberg, E. J., Sedinger, J. S., Nonne, D. V. & Atamian, M. T. Seasonal reproductive costs contribute to reduced survival of female greater sage-grouse. *J. Avian Biol.* **44**, 149–158 (2013).

75. Hadley, G. L., Rotella, J. J. & Garrott, R. A. Evaluation of reproductive costs for Weddell seals in Erebus Bay, Antarctica. *J. Anim. Ecol.* **76**, 448–458 (2007).

76. Sanz-Aguilar, A., Tavecchia, G., Pradel, R., Mínguez, E. & Oro, D. The cost of reproduction and experience-dependent vital rates in a small petrel. *Ecology* **89**, 3195–3203 (2008).

77. Desprez, M. *et al.* Age-specific cost of first reproduction in female southern elephant seals. *Biol. Lett.* **10**, 20140264 (2014).

78. Ergon, T., Yoccoz, N. G. & Nichols, J. D. Estimating Latent Time of Maturation and Survival Costs of Reproduction in Continuous Time from Capture–Recapture Data. in *Modeling Demographic Processes In Marked Populations* (eds. Thomson, D. L., Cooch, E. G. & Conroy, M. J.) 173–197 (Springer US, 2009).

79. Nur, N., Geupel, G. R. & Ballard, G. The use of constant-effort mist-netting to monitor demographic processes in passerine birds: annual variation in survival, productivity, and floaters. in *3rd Partners in Flight workshop, US Department of Agriculture, Forest Service, Rocky Mountain Research Center, Ogden, UT* 185–194 (2000).

80. Regehr, E. V., Lunn, N. J., Amstrup, S. C. & Stirling, I. *Supplemental materials for the analysis of capture-recapture data for polar bears in western Hudson Bay, Canada, 1984-2004*. (US Geological Survey, 2007).

81. Grande, J. M. *et al.* Survival in a long-lived territorial migrant: effects of life-history traits and ecological conditions in wintering and breeding areas. *Oikos* **118**, 580–590 (2009).

82. Lebreton, J.-D., Burnham, K. P., Clobert, J. & Anderson, D. R. Modeling survival and testing biological hypothesis using marked animals: a unified approach with case studies. *Ecol. Monogr.* **62**, 67–118 (1992).

83. Pradel, R., Hines, J. E., Lebreton, J.-D. & Nichols, J. D. Capture-recapture survival models taking account of transients. *Biometrics* **53**, 60–72 (1997).

84. Choquet, R., Lebreton, J.-D., Gimenez, O., Reboulet, A.-M. & Pradel, R. U-CARE: Utilities for performing goodness of fit tests and manipulating CApture-REcapture data. *Ecography* **32**, 1071–1074 (2009).

85. Brawn, J. D., Karr, J. R., Nichols, J. D. & Robinson, W. D. Demography of forest birds in Panama: How do transients affect estimates of survival rates? *Ostrich* **69**, 22 (1998).
